# Real-Life Diagnostic Accuracy of MRI in Prenatal Diagnosis

**DOI:** 10.1155/2020/4085349

**Published:** 2020-09-29

**Authors:** Manuel Recio Rodríguez, Cristina Andreu-Vázquez, Israel J. Thuissard-Vasallo, Raquel Cano Alonso, Carmina Bermejo López, Ines Tamarit Degenhardt, Pilar Martínez Ten

**Affiliations:** ^1^Departamento de Diagnóstico por Imagen, Hospital Universitario Quironsalud Madrid, Madrid, Spain; ^2^Departamento de Medicina, Facultad de Ciencias Biomédicas, Universidad Europea de Madrid, Madrid, Spain; ^3^Delta Ecografía, Madrid, Spain; ^4^Departamento de Obstetricia y Ginecología, Hospital Universitario Quironsalud Madrid, Madrid, Spain

## Abstract

There is some controversy about the value of fetal MRI in prenatal diagnosis, and most of the studies examine its accuracy in central nervous system (CNS) pathology. The objective of this retrospective study was to assess the diagnostic accuracy and usefulness of fetal MRI in the prenatal diagnosis of central nervous system (CNS) pathology and non-CNS pathology. Patients referred to the Radiology Department between 2007 and 2018 for a fetal MRI after detection of an anomaly in the fetal ultrasound, a high-risk pregnancy, or an inconclusive fetal ultrasound (*n* = 623) were included in the study. Postnatal diagnosis was used to assess the diagnostic accuracy of MRI. Fetal MRI was considered to provide additional information over fetal ultrasound when findings of the fetal MRI were not detected in the fetal ultrasound or when established a pathological condition that was not detected in the fetal ultrasound. Fetal MRI provided useful information for the perinatal management and prognosis over fetal ultrasound when findings of the fetal MRI changed the postnatal prognosis, leaded to the decision to legally terminate the pregnancy, changed prenatal or postnatal follow-up, or helped in the planning of prenatal or postnatal treatment. Fetal MRI offered an accurate diagnosis in 97% of cases (compared to 90.4% of fetal ultrasound; *p* < 0.001). Concordance between fetal ultrasound and fetal MRI was 92.1%. Fetal MRI provided additional information over fetal ultrasound in 23.1% of cases. In 11.6% of cases, the information was useful for the perinatal management and prognosis. In 45 cases (7.2%), fetal MRI was the only accurate diagnosis. In conclusion, fetal MRI has a superior diagnostic accuracy, especially in CNS pathology, and provides additional useful information in CNS, thoracic, and abdominal pathology.

## 1. Introduction

Fetal ultrasound has been described as the first-line diagnostic examination to identify prenatal congenital abnormalities [1], and the majority of fetal ultrasound examinations are diagnostic. However, the technique has technical limitations even in experienced hands. Accordingly, in cases where the pathology is complex, an additional approach is needed to confirm or complement the ultrasound findings. Fetal magnetic resonance (MRI) is a complementary diagnostic technique to fetal ultrasound that does not present the same limitations as the latter in cases of advanced gestational age, oligohydramnios, inadequate fetal position, and maternal obesity. Moreover, fetal MRI visualization of the brain is not affected by interposition of the cranial vault [2].

Although MRI does not use ionizing radiation and is assumed to be safe, not enough research has been carried out to prove long-term safety [3]. Therefore, MRI should be performed when the benefit outweighs the potential side effects [3]. All fetuses should have a screening ultrasonogram for the detection of fetal anomalies and pregnancy complications before performing a fetal MRI [4]. Limitations of fetal MRI include the lack of availability of equipment and radiology expertise, higher cost, and longer time to perform an examination [4] and lower spatial resolution than ultrasound at early gestational age because of the small size of the fetus [5]. Additionally, fetal movement may cause the appearance of artifacts [5]. To avoid this, measures are taken such as fasting the mother in the hours before the exam or ensuring that the she is comfortable during the procedure, especially at advanced gestational age. In addition, current MRI software and hardware allow MRI exams to be performed with high-quality images obtained in less than 1 second, allowing fetal imaging without maternal or fetal sedation [4].

As for all patients, there are absolute contraindications to MRI in pregnant women (e.g., a ferromagnetic cerebral aneurysm clip, cardiac pacemaker), and some patients are too claustrophobic to undergo the examination. In addition, the use of intravenous contrast is not accepted because gadolinium contrast freely passes through the placenta. The transferred gadolinium then passes into the fetal circulation where it is excreted into the urinary tract and subsequently into the amniotic fluid. Some of the gadolinium in the amniotic fluid will be reabsorbed by the maternal circulation, while some will be swallowed and reabsorbed through the fetal GI tract [6]. Furthermore, the half-life of gadolinium in the fetus is unknown. Some authors have recently reported the potential nephrotoxicity risk of gadolinium (nephrogenic systemic fibrosis) [7].

Since the first reports of fetal MRI in 1983 [8], the modality has been increasingly used as an adjunct to ultrasound [9], mainly on cerebral pathology. There are fewer publications describing fetal MRI use in chest and abdominal pathology [10], or other less common anatomical locations such as musculoskeletal pathology [11].

While the diagnostic accuracy of fetal ultrasound (compared with postnatal diagnosis or autopsy) has been widely presented, few studies have assessed the accuracy of fetal MRI in the prenatal diagnosis of different anomalies, and most of these examined central nervous system (CNS) pathology. In a meta-analysis of 34 studies [12], including 959 cases of cerebral fetal MRI confirmed by postnatal surgery or autopsy, the diagnostic accuracy of fetal MRI was approximately 91%, 16 percentage higher than that of fetal ultrasound. Likewise, Gonçalves et al. [13] reported that the sensitivity of fetal MRI in non-CNS-related pathology was 80%, as compared with 77.8% for fetal ultrasound.

There is also some controversy about the value of fetal MRI in prenatal diagnosis and its ability to provide additional information over fetal ultrasound and the extent to which the additional information is useful for perinatal management and prognosis, with published studies providing conflicting findings. In a systematic review of prenatal diagnosis of CNS pathology, it was concluded that fetal MRI provided additional information in approximately 22.1% of cases and resulted in a change in clinical management in some 30% [14]. By contrast, Paladini et al. [15] reported that fetal MRI provided clinically relevant additional information in only 7.9% of cases.

Given the disparity in results from different studies, the objective of the present work was to assess the prenatal diagnostic accuracy and usefulness of fetal MRI in CNS and non-CNS pathology in patients with previous fetal ultrasound examinations referred to the Radiology Department.

## 2. Materials and Methods

The study protocol was approved by the Medication Research Ethics Board of the Jimenez Diaz Foundation (code EO006-19_HUQM). All patients signed the written informed consent, which includes permission for the use of their clinical data for research purposes, before the fetal MRI was performed.

### 2.1. Patients

A cohort of pregnant women referred to the diagnostic imaging service of our hospital between January 2007 and December 2018 were studied. In a previous transabdominal fetal ultrasound study (performed and interpreted by qualified gynecologists from 27 different centers), the subjects presented a prenatal anomaly or an indication for fetal MRI for high-risk pregnancy or inconclusive fetal ultrasound.

### 2.2. Interventions

The diagnosis by fetal MRI was made in the 7 days following fetal ultrasound, without sedation after 4 hours fasting. All fetal MRI studies were performed at the same hospital and interpreted by the same radiologist, with more than 15 years of experience.

### 2.3. Fetal MRI Procedure

Fetal MRI was carried out using a Signa HDxt 1.5T MRI Scanner (from 2007 to 2010) and an Optima MR450w 1.5T MRI Scanner (from 2010 to 2018) (General Electric, Milwaukee, WI). An 8-channel torso-array coil was used in the Signa HDxt 1.5T MRI Scanner and a 32-channel cardiac or body coil in the Optima MR450w 1.5T MRI Scanner. In all the studies, single-shot Fast Spin Echo T2 (SSFSE T2) sequences, FIESTA (fast imaging employing steady-state acquisition) balanced sequences, T1 weighted sequences (LAVA—liver acquisition with volume acquisition), T2 gradient echo, and diffusion-weighted imaging were generated. Occasionally, recovery-inversion sequences or a cerebral tractography using diffusion tensor imaging were used. Sequence parameters used are shown in Table 1.

### 2.4. Study Variables and Definitions

Nonpersonal sociodemographic and clinical data were recorded for all patients. Prenatal dates and diagnoses by fetal ultrasound and fetal MRI, gestational age at the time of fetal MRI, and postnatal diagnosis by imaging tests or diagnosis by autopsy were registered. Postnatal diagnosis was made by fetal autopsy in cases of legal termination of pregnancy. In newborns with CNS pathology, postnatal diagnosis was made by ultrasound and MRI. In the cases of thoracic pathology, the postnatal diagnosis was established by chest radiography and/or thoracic computed tomography and, occasionally, thoracic MR. In cases of abdomino-pelvic pathology, ultrasound and, occasionally, MRI were used. In those cases where postnatal surgery was required, the postnatal diagnosis was established during surgery and with the anatomopathological diagnosis of the surgical specimens. Patients with an unknown postnatal diagnosis were excluded from the study.

The following terms were defined. Accurate fetal ultrasound/MRI diagnosis: the prenatal diagnosis by fetal ultrasound/MRI coincides with the postnatal diagnosis or autopsy. Concordant fetal ultrasound and fetal MRI diagnosis: the prenatal diagnosis by fetal ultrasound coincides with the prenatal diagnosis by fetal MRI. Fetal MRI provides additional information over fetal ultrasound: the fetal MRI diagnosis coincides with the fetal ultrasound diagnosis, and findings of the fetal MRI were not detected in the fetal ultrasound, or the fetal MRI established a pathological condition that was not detected in the fetal ultrasound. Fetal MRI provides useful information for perinatal management and prognosis over fetal ultrasound: fetal MRI diagnosis is concordant with fetal ultrasound diagnosis, and the findings of the fetal MRI [1] change the postnatal prognosis, [2] lead to the decision to legally terminate the pregnancy, [3] result in a change in prenatal or postnatal follow-up, or [4] help in the planning of prenatal or postnatal treatment. The fetal MRI is the only accurate diagnosis: fetal MRI diagnosis is accurate and nonconcordant with that of fetal ultrasound (i.e., MRI diagnosis accurate and fetal ultrasound diagnosis inaccurate).

### 2.5. Statistical Analysis

For the descriptive analysis, absolute (*n*) and relative frequencies (%) were used to express the qualitative variables. The parametric behavior of the quantitative variables was verified and variables were described using the mean ± standard deviation (SD) if they followed a normal distribution or using the median (interquartile range (IQR)) if they did not.

To study the statistical significance between the diagnostic accuracy of fetal ultrasound and fetal MRI, and the concordance between both diagnostic techniques in the different indications, Chi-squared tests or Fischer's exact tests were performed. Similarly, the proportion of cases in which fetal MRI provided additional information and useful information for perinatal management and prognosis over fetal ultrasound are described and compared among the different indications. Data were analyzed using the SPSS statistical package, v21.0 (IBM Corp., Armonk, NY). A significance level <5% was used.

## 3. Results

**Figure 1:**
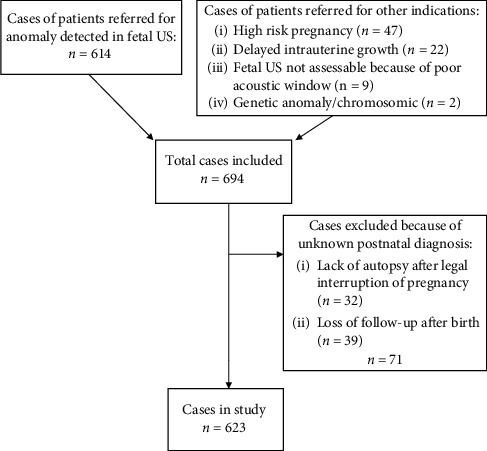
Flow diagram. Cases of patients referred for prenatal diagnosis by magnetic resonance imaging between January 2007 and December 2018.

A total of 694 pregnant women were attended in our hospital for a fetal MRI complementary to fetal ultrasound between January 2007 and December 2018. Diagnostic confirmation was not possible in 71 women because of a lack of postnatal follow-up, and so 623 women were included in the final study (Figure 1).

The median age in the study population on the day of MRI was 35.1 (5.1) years. Median gestational age was 29.4 (8.2) weeks, and in 147 cases (23.6%), gestational age was ≤24 weeks. Forty of the 623 gestations (6.4%) were twins.

Table 2 shows the indications for the 623 cases included in the study. Most of them (429 cases; 68.9%) corresponded to CNS pathology, 37 cases (5.9%) to thoracic pathology, 61 cases (9.8%) to abdominal pathology, and 96 cases (15.4%) to other indications.

### 3.1. Diagnostic Accuracy and Fetal MRI and Fetal Ultrasound Concordance

The diagnostic accuracy of the fetal ultrasound and the fetal MRI and the concordance between the two tests are shown in Table 3. The diagnostic accuracy of the fetal MRI was higher than that of fetal ultrasound in all cases and all indications. The differences in diagnostic accuracy between fetal ultrasound and fetal MRI were significant for all cases (90.4% vs 97%; *p* < 0.001) and for CNS pathology (89.7% vs 98.1%; *p* < 0.001).

Fetal ultrasound and fetal MRI were concordant in 574 of the 623 cases (92.1%). The greatest concordance between diagnoses was recorded in the group of other indications (97.9%), and concordance was less in CNS pathology (90.2%).

The diagnostic accuracy of fetal MRI was not significantly different between cases with gestational age ≤24 weeks and cases with gestational age >24 weeks (97.3% and 96.9%, *p*=1.000). Differences in the diagnostic accuracy of fetal MRI between cases of gestational age less or greater than 24 weeks were not observed for the different indications.

The cases where the diagnosis by fetal MRI and fetal ultrasound did not match with the postnatal diagnosis or autopsy (inaccurate diagnosis) are recorded in Table 4. There were 45 cases in which fetal MRI was the only accurate diagnosis, while only four cases had an accurate diagnosis by fetal ultrasound but an inaccurate diagnosis by fetal MRI. Inaccurate diagnosis by fetal MRI and also by fetal ultrasound occurred in 15 cases.

### 3.2. Additional Information and Useful Information for Perinatal Management and Prognosis of Fetal MRI Over Fetal Ultrasound

**Figure 2:**
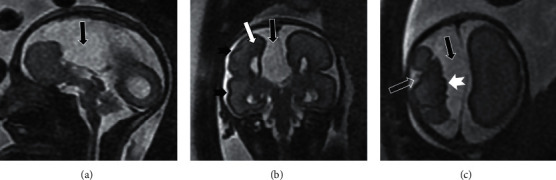
Complete agenesis of corpus callosum. Gestational age: 27 weeks. (a) Sagittal SSFSE T2. (b) Coronal SSFSE T2. (c) Axial SSFSE T2. Complete agenesis of corpus callosum with right interhemispherical cyst with mass effect (solid black arrow) is shown. The right cerebral hemisphere is smaller in size, and malformation of cortical development is evident, with multiple anomalous sulci, bumpy cortical surface (solid black arrowhead), and cortical invaginations (empty white arrow). Regions of associated polymicrogyria (solid white arrowhead) and heterotopias bulging on the ventricle wall are depicted (solid white arrow). Fetal MRI provides useful information for perinatal management and prognosis over fetal ultrasound by detecting anomalies of the cerebral cortex and heterotopies not visible in the fetal ultrasound.

**Figure 3:**
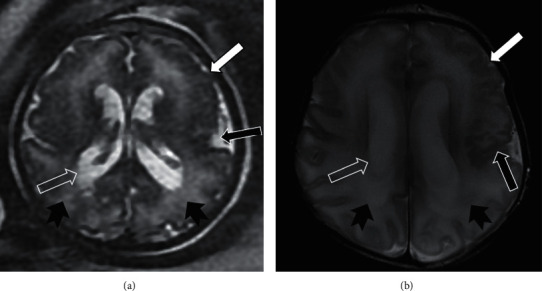
CMV infection. Gestational age: 35 weeks. (a) Fetal Axial SSFSE T2. (b) Postnatal cerebral MRI at 8 days of life, axial FSE T2. The presence of diffuse affectation of the white matter of parietal predominance (solid black arrowhead) with mild ventriculomegaly (empty white arrow) is shown. Fetal MRI provided useful information for perinatal management and prognosis over fetal ultrasound by detecting left frontal (solid white arrow) and left Sylvian fissure (solid black arrow) polymicrogyria not visible in the fetal ultrasound.

**Figure 4:**
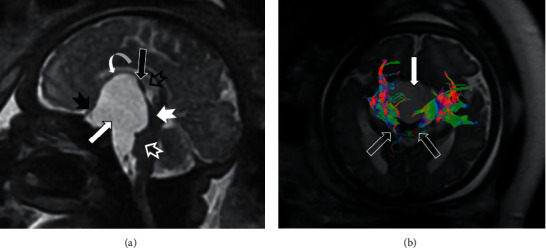
Arachnoid cyst associated with persistence of Liliequist membrane. Gestational age: 36 weeks and 2 days. (a) Sagittal SSFSE T2. Large-sized arachnoid cyst (solid white arrow) anterior to the mesencephalon extending into the prepontine cistern and suprasellar region is shown. The cyst produces a slight mass effect of the protuberance (empty white arrowhead) and an important mass effect on the mesencephalon (solid white arrowhead), hypothalamic region (empty black arrowhead), mammillary bodies (solid black arrow), the floor of the third ventricle (curved white arrow), and optic chiasm (solid black arrowhead). (b) Diffusion tensor with axial tractography image. The cyst (solid white arrow) extends towards the interpeduncular cistern of the mesencephalon and displaces the corticospinal tracts in a posterolateral direction (empty white arrows). Fetal MRI provides additional information because the real extent of the lesion is better evaluated than with ultrasound.

**Figure 5:**
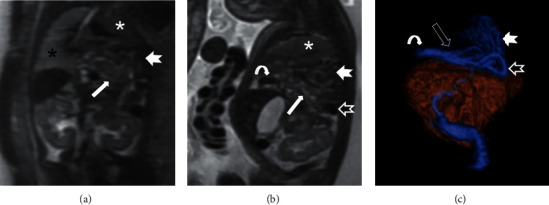
Left diaphragmatic hernia. Gestational age: 27 weeks and 6 days. (a) Coronal SSFSE T2. (b) Sagittal SSFSE T2. (c) Volume rendering coronal reconstruction. Complete herniation of jejunal (solid white arrow) and ileal (solid white arrowhead) loops is observed. Herniation of the hepatic angle of the colon (curved white arrow), ascending colon and cecum (empty white arrow) is also observed, and they are located above the splenic angle of the colon (empty white arrowhead). The left lung is hypoplastic and is collapsed in the left upper hemithorax (white asterisk). The cardiomediastenic displacement towards the right hemithorax and normal right lung (black asterisk) is shown. No hepatic or gastric herniation is observed. Relative pulmonary volume is 72.64%; the intensity ratio of the pulmonary parenchyma relative to the hepatic parenchyma is >1 and the intensity ratio of the pulmonary parenchyma in relation to the CSF is 0.78, implying a good prognosis. Fetal MRI provided useful information for perinatal management and prognosis over the fetal ultrasound.

**Figure 6:**
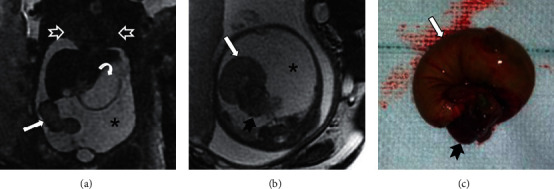
Intestinal volvulus. Gestational age 28 weeks and 2 days. (a) Coronal SSFSE T2. (b) Axial SSFSE T2. (c) Photograph of surgical specimen. Bichorial biamniotic gestation. One of the twins presents with an intestinal volvulus, along with meconial ileus and meconial peritonitis. The presence of massive ascites (black asterisk) that compress both lungs (empty white arrowhead) is shown. The stomach is dilated (curved white arrowhead). Dilated and volvulated ileal loops (solid white arrow) with distal necrosed clustered loops (solid black arrowhead) are shown. Fetal MRI diagnosed intestinal volvulus not described in fetal ultrasound providing useful information for perinatal management and prognosis over fetal ultrasound.

Table 5 shows the proportion of cases in which fetal MRI provides additional information and useful information for perinatal management and prognosis over fetal ultrasound. Fetal MRI provided additional information in 23.1% of cases and useful information for perinatal management and prognosis over fetal ultrasound in 11.6% of cases. Fetal MRI was the only accurate diagnosis in 7.2%. In the CNS pathology indication, fetal MRI was the only accurate diagnosis in 39 of the 429 cases (9.1%) and provided additional information and useful information for perinatal management and prognosis over fetal ultrasound in 96 (22.1%) and 47 cases (11%), respectively (Figures 2–4). The greatest contribution of additional information and useful information for perinatal management and prognosis over fetal ultrasound occurred in cases with thoracic pathology indication (56.8% and 35.1%, respectively), while fetal MRI was the only accurate diagnosis in 2.7% of cases (Figure 5). The abdominal pathology was the second indication in which fetal MRI provided more additional information and useful information for perinatal management and prognosis over fetal ultrasound (27.9% and 11.5%, respectively), and MRI was the only accurate diagnosis in 4.9% of the cases (Figure 6). Overall, fetal MRI provided less additional information and less useful information over fetal ultrasound in cases referred with other indications (10.4% and 5.2%, respectively), and MRI was the only accurate diagnosis in 2.1% of the cases.

### 3.3. Accuracy, Concordance, and Usefulness of Fetal MRI in Cases Referred from Reference and Nonreference Ultrasound Centers

Of the 623 cases, 362 (58.1%) were referred from centers considered to be ultrasound reference centers and 261 (41.9%) from centers considered not to be reference centers. The accuracy of fetal ultrasound was not significantly different between centers (90.1% vs 89.7%; *p*=0.608), but the MRI provided additional information and useful information for perinatal management and prognosis over fetal ultrasound to a greater extent when cases were referred from ultrasound reference centers than from nonreference centers (Table 6).

## 4. Discussion

The present study, based on 623 cases treated during the last 11 years in the Radiology Department, confirms the usefulness of fetal MRI as an adjunct to fetal ultrasound, especially in those cases referred with CNS pathology. In fact, CNS pathology was the most frequent indication in our study, corresponding to 68.9% of cases. The diagnostic accuracy of fetal MRI and fetal ultrasound in the detection of CNS pathology was 98.1% and 89.7%, respectively. These values are slightly higher for fetal MRI and lower for fetal ultrasound than reported by Paladini et al. [15] (94.4% and 91.3%, respectively) and are higher for both techniques than those reported in the meta-analysis of Jarvis et al. [12] (91% and 75%, respectively). In the multicenter MERIDIAN study [16], the values of diagnostic accuracy were 95% for fetal MRI and 82% for fetal ultrasound. The concordance between the fetal MRI and fetal ultrasound diagnosis in the 429 cases described in the present study with indication of CNS pathology was 90.2%, which is slightly higher than that reported by Paladini et al. [15] based on 126 cases (86.5%). In the present study, no significant differences were found in the diagnostic accuracy of fetal MRI between those cases with a gestational age of 24 weeks or less and those with a gestational age above 24 weeks. This contrasts with the study by Paladini et al. [15], who described a diagnostic accuracy of fetal MRI lower than that of fetal ultrasound when the former was carried out before week 24 of gestation (90.4% vs 93.1% for fetal MRI and fetal ultrasound, respectively). Nevertheless, caution should be exercised when interpreting fetal MRI performed before week 24-25 of gestation (10), as several CNS pathologies cannot be accurately diagnosed until the gestational age is greater than 24 weeks, such as the delay in the rotation of the vermis [17], or in the resolution of Blake's pouch cyst [18], which may be mistaken for vermian hypoplasia. When performed before week 24, MRI also has a low sensitivity in the diagnosis of heterotopias [19]. In this regard, the discrepancy between our results and those of Paladini et al. [15] is likely due to the lower number of cases with these pathologies in our study.

Assessing the usefulness of fetal MRI is more controversial, and different studies report highly variable values. Despite the fact that, like fetal ultrasound, fetal MRI is commonly considered a non-operator-dependent technique, both the equipment used to perform the test and the radiologist's experience in interpreting the results in complex pathologies can affect assessment of the usefulness of fetal MRI. Likewise, there is no consensus on the definition of useful information for perinatal management and prognosis over fetal ultrasound, and this also contributes to the variability of the results across the different studies. In our study, fetal MRI contributed 22.4% of additional information over fetal ultrasound, and 11% of useful information for perinatal management and prognosis over fetal ultrasound in cases with CNS pathology, which supports the usefulness of fetal MRI even when the concordance between the diagnoses exceeds 90%. Fetal MRI provided useful information for perinatal management and prognosis over fetal ultrasound in a large number of cases with ischemic-hemorrhagic pathology and tuberous sclerosis, although it was less useful in conditions affecting the corpus callosum, other midline defects, ventriculomegaly, and malformations of the posterior fossa. Similar to our study, Paladini et al. [15] reported that fetal MRI provided clinically relevant information in 7.9% of cases. By contrast, the MERIDIAN study [16] reported that fetal MRI provided additional information over fetal ultrasound in 49% of cases and modified the prognostic information in 20%. The aforementioned study has received some criticism, especially from Paladini et al. in a letter to the Lancet [20], which emphasized the importance of using transvaginal ultrasound with high-resolution transducers in neurosonography studies (not used in the MERIDIAN study) that, presumably, would have reduced the prognostic information provided by fetal MRI.

Thoracic pathology was evident in 5.9% of cases in our study, with pulmonary parenchymatous lesions being the commonest indication. From a clinical perspective, however, the main value of fetal MRI in this clinical scenario is to study congenital diaphragmatic hernia [21], in spite of its low incidence [22]. Fetal MRI provided additional information in 56.8% of the cases and useful information for perinatal management and prognosis over fetal ultrasound in 35.1%. This high figure is mainly due to the 12 cases of congenital diaphragmatic hernia, where fetal MRI could accurately detect the presence of the hernia and the compromised abdominal internal organs, measure the pulmonary volume and its signal, determine the degree of hypoplasia, and quantify the percentage of hepatic herniation, permitting a neonatal prognosis to be established [23–25]. In the study of pulmonary pathology by Levine et al. [26], fetal MRI provided additional information over fetal ultrasound in 38% of cases and resulted in a change in treatment in 8%.

Abdominal pathology corresponded to 9.8% of the cases in our study, in which fetal MRI and fetal ultrasound showed similar accuracies. The fetal MRI provided additional information and useful information for perinatal management and prognosis over fetal ultrasound in 27.9% and 11.5% of the cases. The most frequent cases referred were those with gastrointestinal pathology (28 cases), with fetal MRI providing additional information and useful information for perinatal management and prognosis in 5 and 4, and producing the only accurate diagnosis in one case. The values of additional information and useful information for perinatal management and prognosis over fetal ultrasound are lower than those reported by Manganaro et al. [27] in a prospective study of 38 cases, wherein fetal MRI provided additional information in more than 60% of cases, ruled out the presence of anomalies diagnosed in the fetal ultrasound in 5.2% of cases, and changed the fetal ultrasound diagnosis in 5.2% of cases. Once again, the lack of consensus in the definitions of additional and useful information could explain the discrepancies between the results.

Regarding those cases with indication for urogenital pathology, fetal MRI provided additional over fetal ultrasound in 16.7% of cases. Nonetheless, it is noteworthy that these results are only based on 12 cases. In a study of 46 fetuses with urogenital anomalies, Kajbafzadeh et al. [28] reported that MRI provided additional information in 37% of cases.

Overall, fetal MRI provided additional information in 23.1% of the 623 cases, which corresponded to useful information for perinatal management and prognosis in 11.6%. Fetal MRI was the only accurate diagnostic method in 45 cases (7.2%). To our knowledge, our study represents the largest number of cases describing the value of fetal MRI both in CNS-related and non-related pathologies.

## 5. Conclusions

Fetal MRI has a superior diagnostic accuracy, especially in CNS pathology, and provides additional useful information in CNS, thoracic, and abdominal pathology. The combination of fetal MRI with high-quality ultrasound facilitates additional diagnostic information, which widens the possibilities for detecting early perinatal development disorders.

## Figures and Tables

**Table 1:** Sequence parameters.

	SSFSE T2 sequences	FIESTA balanced sequences	T1-weighted images (3D LAVA)	Diffusion-weighted imaging	Gradient echo T2	Recovery-inversion sequences SSFSE T2	Diffusion tensor
Repetition time (ms)	1583	5.1	6.2	7706	1125	1955	4278
Echo time (ms)	148	Minimum (1.9)	13.1	72.6	21.8	35.7	104.6
Matrix	320 × 320	192 × 256	192 × 192	128 × 128	128 × 192	160 × 160	64 × 64
Field of view (cm)	32	38	38	40	40	40	20
Bandwidth (kHz)	41.7	83.3	83.3	250	250	41.7	167
Number if excitations	0.5	0.71	0.71	8	4	1	2
Slice thickness (mm)	3 (spacing 0.3 mm)	3	3	3 (spacing 0.3 mm)	3 (spacing 0.3 mm)	3 (spacing 0.3 mm)	3 (spacing 0 mm)
Angle (°)		85°	12°				
Inversion time (ms)				180		1500	
B (s/mm^2^)				1000			800
Phase-encoding directions							16

FIESTA: fast imaging employing steady-state acquisition, LAVA: liver acquisition with volume acquisition, SSFSE: single-shot fast spin echo.

**Table 2:** Indications of the 623 cases with a previous ultrasound study referred for fetal MRI between January 2007 and December 2018.

Indication		*n* (%)
*CNS pathology*		**429 (68.9)**
	Ventriculomegaly	148 (34.5)
	Posterior fossa malformations	71 (16.6)
	Abnormalities of the corpus callosum	58 (13.5)
	Other midline anomalies	38 (8.9)
	Infectious pathology	30 (7.0)
	Ischemic/hemorrhagic pathology	25 (5.8)
	Tumoral pathology and lesions of the cranial vault, neck or orbits	24 (5.6)
	Spinal column pathology	12 (2.8)
	Tuberous sclerosis	13 (3.0)
	Malformations of cortical development	10 (2.3)

*Thoracic pathology*		**37 (5.9)**
	Parenchymatous lesions	22 (59.5)
	Congenital diaphragmatic hernia	12 (32.4)
	Mediastenic or pleural pathology	3 (8.1)

*Abdominal pathology*		**61 (9.8)**
	Digestive pathology	28 (45.9)
	Urogenital pathology	12 (19.7)
	Abdominal masses	13 (21.3)
	Abdominal wall defects	4 (6.6)
	Vascular pathology	4 (6.6)

*Other indications*		**96 (15.4)**
	Normal ultrasound in high-risk pregnancy	47 (49.0)
	Intrauterine growth retardation	22 (22.9)
	Ultrasound not valid due to poor acoustic window	9 (9.4)
	Placental pathology	9 (9.4)
	Musculoskeletal pathology	7 (7.3)
	Genetic or chromosomal abnormality	2 (2.1)

**Table 3:** Diagnostic accuracy of fetal ultrasound and fetal magnetic resonance relative to the postnatal diagnosis or autopsy and concordance (*n* = 623).

Indication	Diagnostic accuracy relative to postnatal diagnosis					Concordance between fetal ultrasounsd and MRI *n* (%)	
	Of fetal ultrasound *n* (%)		Of fetal MRI *n* (%)		*p*-value		
*Total*	593	(90.4)	604	(97.0)	<0.001	574	(92.1)
CNS pathology (*n* = 429)	385	(89.7)	421	(98.1)	<0.001	387	(90.2)^*∗*^
Thoracic pathology (*n* = 37)	33	(89.2)	34	(91.9)	1.000	36	(97.3)
Abdominal pathology (*n* = 61)	52	(85.2)	54	(88.5)	0.592	57	(93.4)
Other indications (*n* = 96)	93	(96.9)	95	(99.0)	0.621	94	(97.9)^*∗*^

*p*-value of the Chi-squared test for the differences in diagnostic accuracy of fetal ultrasound (US) and fetal magnetic resonance imaging (MRI) for all indications and for each indication. The asterisk (^*∗*^) indicates a statistically significant difference (Chi-squared test or Fischer's exact test, *p* < 0.05) in the concordance between fetal US and fetal MRI for the different indications.

**Table 4:** Cases with inaccurate diagnosis by fetal ultrasound and/or fetal magnetic resonance imaging (MRI).

Inaccurate diagnosis by fetal ultrasound	Accurate diagnosis by fetal MRI	Postnatal diagnosis	Cases (*n*)
*Total*			45
*CNS pathology*			39
Hypoplasia of corpus callosum	Normal	Normal	7
Partial agenesis of the corpus callosum	Normal	Normal	4
Agenesis of the SP	Normal	Normal	5
Poor visualization of CSP	Polymicrogyria and heterotopias that deform the CSP	Polymicrogyria and heterotopias that deform the CSP	1
Small-sized CSP	Complete agenesis of the corpus callosum and the SP	Complete agenesis of the corpus callosum and the SP	1
Hydranencephaly	Alobar holoprosencephaly	Alobar holoprosencephaly	1
Severe ventriculomegaly and interhemispheric cyst	Semilobar holoprosencephaly	Semilobar holoprosencephaly	1
Ventriculomegaly	Ventriculomegaly and venous infarction.	Ventriculomegaly and venous infarction.	1
Ventriculomegaly	Normal	Normal	4
Lissencephaly	Normal	Normal	1
Closed lip schizencephaly	Normal	Normal	1
Posterior fossa arachnoid cyst	Mega cisterna magna	Mega cisterna magna	2
Dandy walker complex	Mega cisterna magna	Mega cisterna magna	1
Dandy walker complex	PHACE syndrome	PHACE syndrome	1
Inferior vermis hypoplasia	Normal	Normal	1
Posterior fossa mass lesion	Dural sinus malformation and thrombosis	Dural sinus malformation and thrombosis	1
Dandy walker complex	Mega cisterna magna	Mega cisterna magna	1
Microcephaly	Normal	Normal	1
Tethered spinal cord	Normal	Normal	1
Blakes pouch cyst	Mild vermian hypoplasia	Mild vermian hypoplasia	1
Cleft lip	Normal	Normal	2
*Thoracic pathology*			**1**
CPAM	Congenital diaphragmatic hernia	Congenital diaphragmatic hernia	1
*Abdominal pathology*			**3**
Renal cyst	Congenital adrenal cyst	Congenital adrenal cyst	1
Ileal atresia	Normal	Normal	1
Bilateral adrenal hyperplasia	Normal	Normal	1
*Other indications*			**2**
IUGR	Normal	Normal	2

Inaccurate diagnosis by fetal ultrasound	Inaccurate diagnosis by MRI	Postnatal diagnosis	Cases (*n*)

*Total*			**15**
*CNS pathology*			5
Occipital soft-tissue hemangioma	Occipital soft-tissue hemangioma	Fibrosarcoma	1
Hydrocephalus	Hydrocephalus and ischemic sequelae	Hydrocephalus and cerebral toxoplasmosis with intraparenchymal lesion	1
Mild ventriculomegaly	Mild ventriculomegaly	Mild ventriculomegaly and malformation of the outer ear	1
Epidural hematoma	Bone lesion: Sarcoma or fibrous dysplasia	Intraosseous hemangioma	1
Mega cisterna magna and frontal polymicrogyria	Mega cisterna magna frontal polymicrogyria	Mega cisterna magna	1
*Thoracical pathology*			**3**
CPAM type 3	CPAM type 3	Normal (vanishing lesion of the lung)	1
Pulmonary hybrid lesion	Pulmonary hybrid lesión	Type 1 CPAM	1
Left atrial myxoma	Left atrial myxoma	Left atrial hemangioma	1
*Abdominal pathology*			**6**
Perirenal hematoma	Perirenal lymphangioma	Perirenal urinoma without hydronephrosis	1
Mild dilation of ileal loops	Hypoplastic colon syndrome without ileal dilatation	Ileal atresia	1
Esophageal atresia	Esophageal atresia	Esophageal duplication cyst	1
Left adrenal neuroblastoma	Left adrenal neuroblastoma	Infradiaphragmatic bronchopulmonary sequestration	1
Renal cyst	Pyeloureteral junction stenosis	Pyeloureteral junction stenosis and mesoblastic nephroma in calyceal wall	1
*Other indications*			**1**
Placenta increta	Placenta increta	Normal placenta	1

Accurate diagnosis by fetal ultrasound	Inaccurate diagnosis by fetal MRI	Postnatal diagnosis	Cases (*n*)

*Total*			**4**
*CNS pathology*			**3**
Scoliosis and dorsal hemivertebrae	Dorsal scoliosis	Scoliosis and dorsal hemivertebrae	3
*Abdominal pathology*			**1**
Hepatic calcifications	Normal liver	Hepatic calcifications	1

CNS: central nervous system, SP: septum pellucidum, CSP: cavum septum pelludidum, IUGR: intrauterine growth restriction, CPAM: congenital pulmonary airway malformation.

**Table 5:** Usefulness of fetal magnetic resonance as a complementary procedure to fetal ultrasound in the 623 cases referred between January 2007 and December 2018.

Indication	MRI concordant with fetal ultrasound *n* (%)	MRI provides additional information *n* (%)	MRI provides useful information *n* (%)	MRI is the only accurate diagnosis *n* (%)
*Total (n* *=* *623)*	**574 (92.1)**	**144 (23.1)**	**72 (11.6)**	**45 (7.2)**
*CNS pathology (n* *=* *429)*	**387 (90.2)**	**96 (22.4)**	**47 (11.0)**	**39 (9.1)**
Ventriculomegaly	142 (95.9)	22 (14.9)	10 (6.8)	6 (4.1)
Posterior fossa malformations	65 (91.5)	9 (12.7)	4 (5.6)	6 (8.5)
Abnormalities of the corpus callosum	45 (77.6)	17 (29.3)	8 (13.8)	13 (22.4)
Other midline abnormalities	32 (84.2)	10 (26.3)	4 (10.5)	6 (15.8)
Infectious pathology	30 (100.0)	9 (30.0)	3 (10.0)	0 (0.0)
Ischemic/hemorrhagic pathology	23 (92.0)	14 (56.0)	9 (36.0)	2 (8.0)
Tumoral pathology and lesions of the cranial vault, neck, or orbits	21 (87.5)	7 (29.2)	4 (16.7)	3 (12.5)
Spinal column pathology	8 (66.7)	1 (8.3)	1 (8.3)	1 (8.3)
Tuberous sclerosis	13 (100.0)	6 (46.2)	4 (30.8)	0 (0.0)
Malformations of cortical development	8 (80.0)	1 (10.0)	0 (0.0)	2 (20.0)

*Thoracic pathology (n* *=* *37)*	**36 (97.3)**	**21 (56.8)**	**13 (35.1)**	**1 (2.7)**
Parenchymatous lesions	21 (95.5)	9 (40.9)	1 (4.5)	1 (4.5)
Congenital diaphragmatic hernia	12 (100.0)	12 (100.0)	12 (100.0)	0 (0.0)
Mediastenic or pleural pathology	3 (100.0)	0 (0.0)	0 (0.0)	0 (0.0)

*Abdominal pathology (n* *=* *61)*	**57 (93.4)**	**17 (27.9)**	**7 (11.5)**	**3 (4.9)**
Digestive pathology	26 (92.9)	5 (17.9)	4 (14.3)	1 (3.6)
Urogenital pathology	11 (91.7)	2 (16.7)	0 (0.0)	1 (8.3)
Abdominal masses	12 (92.3)	4 (30.8)	1 (7.7)	1 (7.7)
Abdominal wall defects	4 (100.0)	2 (50.0)	0 (0.0)	0 (0.0)
Vascular pathology	4 (100.0)	4 (100.0)	2 (50.0)	0 (0.0)

*Other indications (n* *=* *96)*	**94 (97.9)**	**10 (10.4)**	**5 (5.2)**	**2 (2.1)**
Normal ultrasound in high-risk pregnancy	47 (100.0)	2 (4.3)	1 (2.1)	0 (0.0)
IUGR	20 (90.9)	2 (9.1)	3 (13.6)	2 (9.1)
Ultrasound not assessable (poor acoustic window)	9 (100.0)	3 (33.3)	0 (0.0)	0 (0.0)
Placental pathology.	9 (100.0)	1 (11.1)	0 (0.0)	0 (0.0)
Musculoskeletal pathology	7 (100.0)	2 (28.6)	1 (14.3)	0 (0.0)
Genetic or chromosomal abnormality	2 (100.0)	0 (0.0)	0 (0.0)	0 (0.0)

**Table 6:** Diagnostic accuracy of the fetal ultrasound relative to postnatal diagnosis, concordance, and usefulness of fetal MRI of the 623 cases referred from reference and non-reference ultrasound centers.

	Fetal ultrasound studies performed in centers of reference (*n* = 362)	Fetal ultrasound studies not performed in centers of reference (*n* = 261)	*p*-value
Diagnostic accuracy of fetal ultrasound	329 (90.9%)	234 (89.7%)	0.608
Concordance between fetal ultrasound and MRI	338 (93.4%)	236 (90.4%)	0.177
MRI provides additional information over fetal ultrasound	96 (26.5%)	48 (18.4%)	0.018
MRI provides additional information useful for perinatal management and prognosis over fetal ultrasound	53 (14.6%)	19 (7.3%)	0.004
MRI is the only accurate diagnosis	22 (6.1%)	23 (8.8%)	0.193

*p*-value of the chi-squared tests for the differences in diagnostic accuracy of fetal ultrasound, concordance, and usefulness of MRI between cases referred from ultrasound reference and nonreference centers.

## Data Availability

The data used to support the findings of this study are available from the corresponding author upon request.

## References

[1] Coakley F. V., Glenn O. A., Qayyum A., Barkovich A. J., Goldstein R., Filly R. A. (2004). Fetal MRI:A developing technique for the developing patient. *American Journal of Roentgenology*.

[2] Levine D. (2001). Ultrasound versus magnetic resonance imaging in fetal evaluation. *Topics in Magnetic Resonance Imaging*.

[3] Valevičienė N. R., Varytė G., Zakarevičienė J., Kontrimavičiūtė E., Ramašauskaitė D., Rutkauskaitė-Valančienė D. (2019). Use of magnetic resonance imaging in evaluating fetal brain and abdomen malformations during pregnancy. *Medicina (Kaunas)*.

[4] Reddy U. M., Filly R. A., Copel J. A. (2008). Prenatal imaging. *Obstetrics & Gynecology*.

[5] Glenn O. A., Barkovich A. J. (2006). Magnetic resonance imaging of the fetal brain and spine: an increasingly important tool in prenatal diagnosis, part 1. *AJNR. American Journal of Neuroradiology*.

[6] Salomon L. J., Siauve N., Balvay D. (2005). Radiology imaging with contrast agents in a mouse model 1. *Radiology*.

[7] Grobner T., Prischl F. C. (2007). Gadolinium and nephrogenic systemic fibrosis. *Kidney International*.

[8] Smith F., Adam A. H., Phillips W. D. (1983). NMR imaging in pregnancy. *The Lancet*.

[9] Yagel S., Cohen S. M., Porat S. (2015). Detailed transabdominal fetal anatomic scanning in the late first trimester versus the early second trimester of pregnancy. *Journal of Ultrasound in Medicine*.

[10] Manganaro L., Antonelli A., Bernardo S. (2018). Highlights on MRI of the fetal body. *La Radiologia Medica*.

[11] Nogueira R. d. A., Werner Júnior H., Daltro P., Lima G. M., Barbosa A. D., Araujo Júnior E. (2018). The role of a novel magnetic resonance imaging sequence in the evaluation of the fetal skeleton: a pilot study. *Radiologia Brasileira*.

[12] Jarvis D., Mooney C., Cohen J. (2017). A systematic review and meta-analysis to determine the contribution of mr imaging to the diagnosis of foetal brain abnormalities in Utero. *European Radiology*.

[13] Gonçalves L. F., Lee W., Mody S., Shetty A., Sangi-Haghpeykar H., Romero R. (2016). Diagnostic accuracy of ultrasonography and magnetic resonance imaging for the detection of fetal anomalies: a blinded case-control study. *Ultrasound in Obstetrics & Gynecology*.

[14] Rossi A. C., Prefumo F. (2014). Additional value of fetal magnetic resonance imaging in the prenatal diagnosis of central nervous system anomalies: a systematic review of the literature. *Ultrasound in Obstetrics & Gynecology*.

[15] Paladini D., Quarantelli M., Sglavo G. (2014). Accuracy of neurosonography and MRI in clinical management of fetuses referred with central nervous system abnormalities. *Ultrasound in Obstetrics & Gynecology*.

[16] Griffiths P. D., Bradburn M., Campbell M. J. (2017). Use of MRI in the diagnosis of fetal brain abnormalities in utero (MERIDIAN): a multicentre, prospective cohort study. *The Lancet*.

[17] Pinto J., Paladini D., Severino M. (2016). Delayed rotation of the cerebellar vermis: a pitfall in early second-trimester fetal magnetic resonance imaging. *Ultrasound in Obstetrics & Gynecology*.

[18] Robinson A. J. (2014). Inferior vermian hypoplasia—preconception, misconception. *Ultrasound in Obstetrics & Gynecology*.

[19] Glenn O. A., Cuneo A. A., Barkovich A. J., Hashemi Z., Bartha A. I., Xu D. (2012). Malformations of cortical development: diagnostic accuracy of fetal MR imaging. *Radiology*.

[20] Paladini D., Malinger G., Pilu G., Timor-Trisch I., Volpe P. (2017). The MERIDIAN trial: caution is needed. *The Lancet*.

[21] Johnson A. M., Hubbard A. M. (2004). Congenital anomalies of the fetal/neonatal chest. *Seminars in Roentgenology*.

[22] Butler N., Claireaux A. E. (1962). Congenital diaphragmatic hernia as a cause of perinatal mortality. *The Lancet*.

[23] Yamoto M., Iwazaki T., Takeuchi K. (2018). The fetal lung-to-liver signal intensity ratio on magnetic resonance imaging as a predictor of outcomes from isolated congenital diaphragmatic hernia. *Pediatric Surgery International*.

[24] Ruano R., Lazar D. A., Cass D. L. (2014). Fetal lung volume and quantification of liver herniation by magnetic resonance imaging in isolated congenital diaphragmatic hernia. *Ultrasound in Obstetrics & Gynecology*.

[25] Weis M., Hoffmann S., Henzler C. (2018). Isolated impact of liver herniation on outcome in fetuses with congenital diaphragmatic hernia—a matched-pair analysis based on fetal MRI relative lung volume. *European Journal of Radiology*.

[26] Levine D., Barnewolt C. E., Mehta T. S., Trop I., Estroff J., Wong G. (2003). Fetal thoracic abnormalities: MR imaging. *Radiology*.

[27] Manganaro L., Saldari M., Bernardo S. (2015). Role of magnetic resonance imaging in the prenatal diagnosis of gastrointestinal fetal anomalies. *La Radiologia Medica*.

[28] Kajbafzadeh A.-M., Payabvash S., Sadeghi Z. (2008). Comparison of magnetic resonance urography with ultrasound studies in detection of fetal urogenital anomalies. *Journal of Pediatric Urology*.

